# Intentional tanning behaviors among undergraduates on the United States’ Gulf Coast

**DOI:** 10.1186/s12889-018-5345-5

**Published:** 2018-04-03

**Authors:** Casey L. Daniel, Natalie R. Gassman, Alyssa M. Fernandez, Sejong Bae, Marcus C. B. Tan

**Affiliations:** 1 Division of Oncological Sciences, University of South Alabama Mitchell Cancer Institute, 1660 Springhill Avenue, Mobile, AL 36604 USA; 2 Division of Preventive Medicine, Department of Medicine, University of Alabama at Birmingham, 1717 11th Avenue South, Birmingham, AL 35205 USA; 3 Comprehensive Cancer Center, University of Alabama at Birmingham, Birmingham, AL USA; 4 Department of Surgery, University of South Alabama College of Medicine, 2451 Fillingim Street, Mobile, AL 36617 USA

**Keywords:** Adolescent, Young adult, Tanning, Skin cancer, Melanoma, Prevention

## Abstract

**Background:**

Rates of melanoma have dramatically increased among adolescents and young adults in recent years, particularly among young women. Exposure to ultraviolet radiation from intentional tanning practices is likely a major contributor to this epidemic. Southern and coastal regions have higher melanoma mortality rates among non-Hispanic whites in other parts of the U.S., yet little is known about tanning practices of adolescents and young adults in these regions. This study determines the prevalence and methods of intentional tanning utilized by an undergraduate population located on the United States’ Gulf Coast.

**Methods:**

Undergraduate students enrolled at a university on the Gulf Coast completed an online survey from March–April 2016, self-reporting their engagement, knowledge, and attitudes regarding outdoor tanning (OT), indoor tanning (IT) and spray tanning (ST). Univariate and multivariate analyses were performed to identify factors associated with tanning behaviors.

**Results:**

2668 undergraduates completed the survey. Of these, 64.9% reported OT tanning, 50.7% reported ever IT, and 21.2% reported ever ST.

**Conclusions:**

In the largest study to date of intentional tanning behaviors of adolescents and young adults from coastal regions, we found high rates of intentional tanning behaviors. There was also significant engagement in spray tanning by this population, not previously reported for adolescents and young adults in a sample of this size. We also identified a high association between different tanning methods, indicating this population engages in multiple tanning behaviors, a phenomenon whose health consequences are not yet known.

## Background

The incidence of skin cancers, especially melanoma, continues to rise, particularly among adolescents and young adults (AYA) [1–3]. The most significant and modifiable risk factor for the development of cutaneous malignancies is ultraviolet (UV) radiation exposure [1, 4–8]. In the AYA population, recreational UV exposure (tanning) is a popular activity [9–15], driven primarily by the perceived social desirability of a tanned appearance [16–20]. The most common methods of tanning are outdoor tanning (OT) and indoor tanning (IT), though non-UV based tanning methods such as spray tanning (ST) are increasingly popular, since they are promoted as a safer alternative to OT and IT [13].

In recent years, numerous studies have examined tanning behaviors in college students. However, these have mostly focused on colleges in the Northeastern and Midwestern regions of the U.S. [9, 10, 21–25]. None of the studies at Southern U.S. colleges have examined all three tanning modalities and their relative frequencies [12, 16, 26, 27]. Developing a comprehensive understanding of tanning behaviors of Southern college students is important for a number of reasons: skin cancer risk increases with the North-South UV index gradient [28]; Southern states receive the highest amount of UV radiation reaching the earth’s surface [28], individuals residing in Southern and coastal regions have the highest mortality rates among non-Hispanic whites [29], national high school student data suggests that tanning behaviors are highest in the South compared with other geographies [30], and proximity to beaches in Southern states has been noted as a motivator for selecting a college along the coast [26]. Therefore, this study sought to investigate all types of intentional tanning behaviors at a coastal university in the South.

## Methods

### Participants and survey administration

Participants were undergraduate students enrolled at a public, state university in Mobile, Alabama. Eligibility criteria for study participation were: age 18 years or older and enrollment at the participating institution. Institutional Review Board approval was obtained prior to data collection. All students enrolled at the institution were sent an email to their university email addresses containing a brief description and link to the survey in March 2016. Subsequent reminders were sent once per week for the following three weeks. Research Electronic Data Capture (REDCap) was used to create a survey and data collection. Students were given a written explanation of informed consent detailing the anonymity of participation upon initiation of the electronic survey. The first 2000 students to complete the survey received an incentive of a $5 USD gift card to Amazon.com, which was distributed electronically.

### Outcome measures

Primary outcome measures were self-reported current OT and/or ever use of: a) IT; and/or b) ST. OT was defined by questions asking if the respondent tanned outdoors year round and/or in what seasons. IT was defined by the question, “Have you ever used a tanning bed before?” ST was defined by the question, “Have you ever gotten a spray tan?” Our explanatory measures included *demographics* (age, college year, sex, race, and family melanoma history); *Fitzpatrick skin type* (scores determined by responses to natural hair color, natural eye color, color of untanned skin, number of freckles, burn tendency, and tan tendency) [31]; *attitudinal variables*; and *risk perceptions*. Attitudinal and risk perception questions were not included in the current analysis.

For race, categories: American Indian or Alaska native; Asian; Hispanic or Latino; Other; and multiracial were combined to form the “Other” option due to small sample sizes and categorized as: ‘White,’ ‘Black or African American,’ and ‘Other.’ Fitzpatrick categories were also combined due to low sample sizes in each response and categorized as: ‘Fitzpatrick Type I/II,’ ‘Fitzpatrick Type III,’ ‘Fitzpatrick Type IV,’ ‘Fitzpatrick Type V/VI.’

### Statistical analysis

Frequencies and percentages were calculated for categorical variables. Binary logistic regressions were utilized to examine relationships between demographic and aforementioned characteristics and the tanning behaviors of interest (OT, IT, and ST). We estimated both unadjusted and adjusted odds ratios (OR) and their corresponding 95% confidence intervals (95% CI) using logistic regression analyses. Multicollinearity of covariates was assessed using the variance inflation factor threshold of 5. Statistical analyses were performed using SAS v9.4, where statistical significance was considered if *P*-values were less than 0.05.

## Results

Of the 10,880 undergraduates contacted, 2668 students completed the survey (24.5% response rate, comparable to other electronic surveys) [32, 33]. The majority of respondents were female (69.3%) and White (68.9%). These respondents reflect the larger campus population, of which 61.5% are female and 61.1% are White. Most participants were upperclassmen: freshman (20.8%); sophomore (22.1%); junior (27.1%); senior (30.0%). Rates of response by class year were also comparable to the university’s enrollment: freshman (28.4%); sophomore (19.7%); junior (20.9%); senior (31.0%). With respect to tanning behaviors, 64.9% reported current OT tanning, 50.7% reported ever IT, and 21.2% reported ever ST. Thirty percent of respondents self-identified as current IT. Demographic and other behavioral characteristics can be found in Table 1. Association between each tanning behavior and demographic and other behavioral characteristics are reported below.

**Table 1:** Demographic characteristics for undergraduate students (*n* = 2668)

Variable	*N* (%)
Age	
18 years old	254 (9.5)
19 years old	509 (19.1)
20 years old	455 (17.1)
21 years old	410 (15.4)
22+ years old	1040 (39.0)
College Year	
Freshman	555 (20.8)
Sophomore	589 (22.1)
Junior	723 (27.1)
Senior	801 (30.0)
Sex	
Male	818 (30.7)
Female	1844 (69.3)
Race	
White	1838 (68.9)
Black or African American	480 (18.0)
Other	350 (13.1)
Alabama resident	
Yes	2119 (79.6)
No	544 (20.4)
Immediate family member with history of melanoma	
No	2015 (75.6)
Yes	184 (6.9)
Don’t know/Not sure	465 (17.5)
Fitzpatrick score	
Fitzpatrick Type I/II	395 (14.8)
Fitzpatrick Type III	780 (29.3)
Fitzpatrick Type IV	875 (32.8)
Fitzpatrick Type V/VI	617 (23.1)
Number of lifetime blistering sunburns	
None	955 (35.8)
1–2	1030 (38.7)
3–5	508 (19.1)
6 or more	172 (6.5)
Outdoor tanner	
No	936 (35.1)
Yes	1732 (64.9)
Ever spray tanned	
No	2033 (78.2)
Yes	566 (21.2)
Ever indoor tanned	
No	1236 (47.7)
Yes	1353 (50.7)
Currently use tanning beds	
No	1054 (69.6)
Yes	461 (30.4)
Intend to use tanning bed next month	
No	2312 (87.1)
Yes	343 (12.9)
Intend to use tanning bed next 12 months	
No	2208 (83.2)
Yes	447 (16.8)
Preferred method of tanning if time, convenience, and price not factors	
Tanning beds	105 (23.4)
Tanning outdoors	267 (59.5)
Spray tan	72 (16.0)
Other	5 (1.1)

### Outdoor tanning

In the sample, 1732 individuals (64.9%) reported OT. On multivariate analysis (Table 2), being female (OR = 1.75, 95% CI [1.39, 2.20]); lifetime history of 1–2 blistering sunburns (AOR = 1.38, 95% CI [1.06, 1.80]); ever having ST (AOR = 2.54, 95% CI [2.03, 3.18]); ever having IT (AOR = 2.54, 95% CI [2.03, 3.18]); and intending to use tanning beds in the next 12 months (AOR = 10.63, 95% CI [5.38, 20.98]) were associated with increased likelihood of OT. Being Black/African American race (adjusted odds ratio [AOR] = 0.05, 95% confidence interval [CI] [0.03, 0.07]); “Other” race (AOR = 0.42, 95% CI [0.30, 0.58]); Don’t know/Not sure about immediate family history of melanoma (OR = 0.67; 95% CI [0.51, 0.88]); Fitzpatrick skin type I/II (AOR = 0.19, 95% CI [0.13, 0.29]); and Fitzpatrick skin type III (OR = 0.59, 95% CI [0.40, 0.86] were associated with decreased likelihood of OT. Age, college year, and state of residence were not significantly associated with OT.

**Table 2:** Variables associated with self-reported outdoor tanning behavior among undergraduate students (*n* = 1732)

Variable	Outdoor Tans *N* (%)	Odds Ratio	95% CI	Adjusted Odds Ratio	95% CI
Age					
18 years old (ref)	156 (9.0)	1.00		1.00	
19 years old	324 (18.7)	1.10	0.81–1.50	1.03	0.67–1.60
20 years old	298 (17.2)	1.19	0.87–1.64	0.86	0.50–1.48
21 years old	303 (17.5)	1.78	1.27–2.49	1.33	0.74–2.41
22+ years old	651 (37.6)	1.05	0.79–1.39	0.58	0.33–1.00
Year					
Freshman (ref)	336 (19.4)	1.00		1.00	
Sophomore	381 (22.0)	1.19	0.94–1.52	1.15	0.78–1.69
Junior	471 (27.2)	1.22	0.97–1.53	0.97	0.62–1.53
Senior	544 (31.4)	1.38	1.10–1.73	1.33	0.82–2.14
Sex					
Male (ref)	454 (26.3)	1.00		1.00	
Female	1274 (73.7)	1.79	1.51–2.12	1.75	1.39–2.20
Race					
White (ref)	1428 (82.4)	1.00		1.00	
Black or African American	85 (4.9)	0.06	0.05–0.08	0.05	0.03–0.07
Other	219 (12.6)	0.48	0.38–0.61	0.42	0.30–0.58
Alabama Resident					
Yes (ref)	383 (22.2)	1.00		1.00	
No	1345 (77.8)	0.73	0.60–0.90	0.79	0.61–1.03
Immediate family member with history of melanoma					
No (ref)	1324 (76.5)	1.00		1.00	
Yes	130 (7.5)	1.26	0.90–1.75	0.68	0.45–1.03
Don’t know/Not sure	277 (16.0)	0.77	0.63–0.95	0.67	0.51–0.88
Fitzpatrick score					
Fitzpatrick Type V/VI (ref)	280 (16.2)	1.00		1.00	
Fitzpatrick Type I/II	225 (13.0)	1.60	1.23–2.08	0.19	0.13–0.29
Fitzpatrick Type III	612 (35.3)	4.33	3.42–5.49	0.59	0.40–0.86
Fitzpatrick Type IV	615 (35.5)	2.84	2.28–3.53	0.88	0.64–1.22
Number of lifetime blistering sunburns					
None (ref)	496 (28.6)	1.00		1.00	
1–2	764 (44.1)	2.66	2.20–3.21	1.38	1.06–1.80
3–5	360 (20.8)	2.25	1.79–2.83	1.07	0.77–1.48
6 or more	112 (6.5)	1.73	1.23–2.42	0.87	0.55–1.37
Ever spray tanned					
No (ref)	1178 (69.8)	1.00		1.00	
Yes	510 (30.2)	6.61	4.95–8.83	2.26	1.60–3.20
Ever used a tanning bed					
No (ref)	572 (34.0)	1.00		1.00	
Yes	1111 (66.0)	5.33	4.46–6.37	2.54	2.03–3.18
Intend to use tanning bed next 12 months					
No (ref)	1287 (74.7)	1.00		1.00	
Yes	437 (25.3)	31.26	16.61–58.84	10.63	5.38–20.98

### Indoor tanning

Of the respondents, 1353 (50.7%) reported ever IT. On multivariate analysis (Table 3), females and Alabama residents were also significantly more likely to have ever IT (AOR = 1.65, 95% CI [1.34, 2.04] and AOR = 1.44, 95% CI [1.14, 1.83], respectively). Lifetime history of blistering sunburns was also associated with greater likelihood of ever IT: reported history of 3–5 burns (AOR = 1.36, 95% CI [1.01, 1.83]) and 6+ burns (AOR = 2.06, 95% CI [1.33, 3.20]). Also associated with increased likelihood of ever IT were: self-reported OT (AOR = 2.47, 95% CI [1.97, 3.10]); ever ST (AOR = 3.34, 95% CI [2.51, 4.45]); and intending to IT in the next 12 months (AOR = 22.82, 95% CI [12.85, 40.54]). Black/African American race and “Other” race were significantly associated with *decreased* likelihood of ever IT (AOR = 0.56, 95% CI [0.39, 0.80] and AOR = 0.68, 95% CI [0.50, 0.94], respectively). Participants reporting, “Don’t know/Not sure,” of an immediate family history of melanoma also demonstrated decreased likelihood of ever having IT (AOR = 0.76, 95% CI [0.59, 0.98]). Participants 22+ years old were 71% more likely to have ever IT (AOR = 1.71, 95% CI [1.02, 2.86]). College year and Fitzpatrick skin type were not significantly associated with IT history in the multivariate analysis.

**Table 3:** Variables associated with self-reported ever indoor tanning among undergraduate students (*n* = 1353)

Variable	Ever indoor tanned *N* (%)	Odds Ratio	95% CI	Adjusted Odds Ratio	95% CI
Age					
18 years old (ref)	104 (7.7)	1.00		1.00	
19 years old	216 (16.0)	1.05	0.77–1.44	0.97	0.64–1.48
20 years old	211 (15.6)	1.27	0.92–1.74	0.87	0.52–1.46
21 years old	219 (16.2)	1.62	1.18–2.24	1.06	0.61–1.84
22+ years old	603 (44.6)	2.00	1.51–2.66	1.71	1.02–2.86
Year					
Freshman (ref)	224 (16.6)	1.00		1.00	
Sophomore	272 (20.1)	1.30	1.02–1.65	1.22	0.85–1.75
Junior	386 (28.5)	1.70	1.35–2.13	1.43	0.93–2.18
Senior	471 (34.8)	2.11	1.69–2.64	1.34	0.86–2.09
Sex					
Male (ref)	294 (21.8)	1.00		1.00	
Female	1055 (78.2)	2.40	2.02–2.86	1.65	1.34–2.04
Race					
White (ref)	1111 (82.1)	1.00		1.00	
Black or African American	106 (7.8)	0.19	0.15–0.24	0.56	0.39–0.80
Other	136 (10.1)	0.43	0.34–0.54	0.68	0.50–0.94
Alabama Resident					
No (ref)	252 (18.7)	1.00		1.00	
Yes	1099 (81.3)	1.24	1.03–1.51	1.44	1.14–1.83
Immediate family member with history of melanoma					
No (ref)	1032 (76.3)	1.00		1.00	
Yes	118 (8.7)	1.65	1.20–2.26	1.01	0.69–1.49
Don’t know/Not sure	203 (15.0)	0.73	0.59–0.90	0.76	0.59–0.98
Fitzpatrick score					
Fitzpatrick Type V/VI (ref)	205 (15.2)	1.00		1.00	
Fitzpatrick Type I/II	208 (15.4)	2.29	1.76–2.96	0.83	0.56–1.23
Fitzpatrick Type III	477 (35.3)	3.11	2.49–3.89	1.06	0.76–1.48
Fitzpatrick Type IV	463 (34.2)	2.26	1.82–2.81	1.13	0.85–1.50
Number of lifetime blistering sunburns					
None (ref)	368 (27.2)	1.00		1.00	
1–2	571 (42.2)	2.00	1.67–2.40	1.17	0.92–1.49
3–5	305 (22.5)	2.40	1.92–3.07	1.36	1.01–1.83
6 or more	109 (8.1)	3.00	2.11–4.26	2.06	1.33–3.20
Outdoor tanner					
No (ref)	242 (17.9)	1.00		1.00	
Yes	1111 (82.1)	5.33	4.46–6.37	2.47	1.97–3.10
Ever spray tanned					
No (ref)	870 (64.3)	1.00		1.00	
Yes	483 (35.7)	7.70	6.00–9.87	3.34	2.51–4.45
Intend to use tanning bed next 12 months					
No (ref)	925 (68.4)	1.00		1.00	
Yes	428 (31.6)	43.53	24.90–76.07	22.82	12.85–40.54

### Spray tanning

In the study sample, 566 participants (21.2%) reported ever ST. On multivariate analysis (Table 4), characteristics significantly associated with increased likelihood of ST were: female sex (AOR = 8.79, 95% CI [5.86, 13.18]; Fitzpatrick skin type I/II (AOR = 1.78, 95% CI [1.11, 2.86]); self-reported OT (AOR = 2.51, 95% CI [1.76, 3.57]); ever IT (AOR = 3.27, 95% CI [2.45, 4.37]); and intending to IT in the next 12 months (AOR = 2.04, 95% CI [1.57, 2.65]). Only Black/African American race was associated with decreased likelihood of ST (AOR = 0.35, 95% CI [0.19, 0.64]). Characteristics not associated with ST included: age, college year, state residence, immediate family history of melanoma, and number of lifetime blistering sunburns.

**Table 4:** Variables associated with self-reported spray tanning among undergraduate students (*n* = 566)

Variable	Ever spray tanned *N* (%)	Odds Ratio	95% CI	Adjusted Odds Ratio	95% CI
Age					
18 years old (ref)	45 (8.0)	1.00		1.00	
19 years old	97 (17.1)	1.09	0.74–1.62	1.42	0.86–2.34
20 years old	94 (16.6)	1.22	0.82–1.81	1.52	0.81–2.83
21 years old	94 (16.6)	1.37	0.92–2.04	1.32	0.67–2.59
22+ years old	236 (41.7)	1.36	0.95–1.94	1.31	0.69–2.49
Year					
Freshman (ref)	102 (18.0)	1.00		1.00	
Sophomore	110 (19.4)	1.03	0.76–1.39	0.74	0.47–1.15
Junior	146 (25.8)	1.13	0.85–1.49	0.76	0.45–1.31
Senior	208 (36.7)	1.55	1.18–2.02	1.14	0.66–1.99
Sex					
Male (ref)	28 (5.0)	1.00		1.00	
Female	536 (95.0)	11.54	7.81–17.05	8.79	5.86–13.18
Race					
White (ref)	503 (88.9)	1.00		1.00	
Black or African American	17 (3.0)	0.10	0.06–0.16	0.35	0.19–0.64
Other	46 (8.1)	0.41	0.30–0.57	0.79	0.53–1.19
Alabama Resident					
No (ref)	116 (20.5)	1.00		1.00	
Yes	449 (79.5)	0.98	0.78–1.24	0.97	0.74–1.28
Immediate family member with history of melanoma					
No (ref)	428 (75.6)	1.00		1.00	
Yes	53 (9.4)	1.48	1.05–2.07	1.05	0.71–1.55
Don’t know/Not sure	85 (15.0)	0.83	0.64–1.08	0.94	0.69–1.27
Fitzpatrick score					
Fitzpatrick Type V/VI (ref)	59 (10.4)	1.00		1.00	
Fitzpatrick Type I/II	114 (20.1)	3.85	2.72–5.45	1.78	1.11–2.86
Fitzpatrick Type III	217 (38.3)	3.55	2.60–4.85	1.29	0.85–1.94
Fitzpatrick Type IV	176 (31.1)	2.36	1.72–3.24	1.18	0.79–1.75
Number of lifetime blistering sunburns					
None (ref)	122 (21.6)	1.00		1.00	
1–2	250 (44.2)	2.18	1.72–2.77	1.14	0.86–1.52
3–5	147 (26.0)	2.78	2.12–3.65	1.33	0.95–1.87
6 or more	47 (8.3)	2.66	1.80–3.92	1.32	0.82–2.13
Outdoor tanner					
No (ref)	56 (9.9)	1.00		1.00	
Yes	510 (90.1)	6.61	4.95–8.83	2.51	1.76–3.57
Ever used a tanning bed					
No (ref)	83 (14.7)	1.00		1.00	
Yes	483 (85.3)	7.70	6.00–9.88	3.27	2.45–4.37
Intend to use tanning bed next 12 months					
No (ref)	343 (60.6)	1.00		1.00	
Yes	223 (39.4)	5.41	4.35–6.74	2.04	1.57–2.65

## Discussion

The current study fills a significant gap in existing literature by examining multiple intentional tanning behaviors among a large population of undergraduates at a Southern, coastal university. While OT is a common tanning behavior, the rates and prevalence of OT are not well investigated in the literature, with varying categorizations of OT making comparisons between studies difficult. However, OT rates are higher in the current population (64.9%) than those reported previously (34.4% among 576 college students in Brooklyn, New York), likely due to proximity of the university to the beach [34]. IT rates of this study population (52.3%) were comparable with other reported rates of undergraduate IT, including an international systematic review finding IT rates of 55% among university students [15]. Sunless tanning product use has only been more recently examined in the literature, and comparisons are challenging because categories of these products can include both over-the-counter lotions and ST. Use of these products, particularly among adolescents and young adults have not been well-defined or investigated. In a 2004 national survey, 11% of U.S. adults claimed to have ever used sunless tanning products [35], while a more recent study examining the use of all sunless tanning products estimated use to be 25% among female teens (12–18) in the U.S. [36]. The current survey’s rate of 21.2% is consistent with the usage among teens and provides a new benchmark for adolescents and young adults.

### Outdoor tanning

As expected, results demonstrated that females are more likely to engage in OT than their male counterparts. This is not surprising given that females, particularly in this age range, are more likely than males to engage in all types of tanning behaviors, as previous studies have demonstrated [12, 21, 34]. Whites were also more likely to engage in OT than other races. This is supported by previous findings demonstrating greater OT rates among Whites compared with non-Whites [34]. Haluza et al. found that individuals with higher Fitzpatrick scores demonstrated lower motives to tan, likely due to their naturally darker skin tones [37]. Individuals with fairer skin may be more inclined to OT in the attempt to achieve the socially perceived attractiveness associated with tanned skin. This may explain the additional finding that lifetime history of 1–2 sunburns was associated with greater likelihood of OT, as fairer skinned individuals are more likely to burn when exposed to UV radiation. More significantly, we found associations between tanning behaviors. Individuals who had ever ST were more than twice as likely to OT and individuals who had ever IT were 2.5 times as likely to OT. Further, those who reported the intention to IT in the next 12 months were over 10 times as likely to OT. These findings suggest there is a significant population of individuals that combine tanning behaviors, and the behaviors of this novel group of intentional combination tanners are not well understood.

### Indoor tanning

We found that females were significantly more likely to IT than males, consistent with previous studies [12, 15, 21, 34], and that Whites were significantly more likely to IT than those of other races [38]. We also found that an increased number of blistering sunburns was proportionally associated with increased likelihood of IT, consistent with previous reports [39, 40]. Additionally, an intention to IT in the next 12 months was strongly associated with history of IT. Interestingly, the results here demonstrate that those students who categorized themselves as residents of the state of Alabama were 1.4 times more likely to IT than non-Alabama residents, indicating potential sociocultural norms contributing to these students’ tanning behaviors. Other types of tanning, including OT and ever ST were also significantly associated with ever IT, indicating again that there is an unexplored population of adolescents and young adults that engage in a combination of tanning behaviors.

### Spray tanning

Research specifically on ST is sparse, though use of non-UV methods for tanning needs to be explored in adolescent and young adult populations. Interventions in recent years have attempted to promote sunless tanning as an alternative to UV (OT or IT) tanning [41, 42], and adoption of these practices among adolescents and young adults needs to be established to gauged the success of these efforts. Our results are consistent with reports of sunless tanning product use among teens 12–18 [36] and combination tanning, as reported in a study of 4601 individuals ages 18–34 years old [35], but this is the first report of ST usage among a large adolescent and young adult population. Females in this study were much more likely to engage in ST than males. Individuals with fairer skin tone and more prone to burning were also more likely to engage in ST, echoing previous research demonstrating an association between fair skin and sunless tanning product use [43]. This finding is likely related to negative experiences or low expectations regarding UV (or IT, specifically) tanning. Again, OT and IT behaviors were associated with a history of ST.

There was significant engagement in multiple tanning behaviors, or combination tanning, amongst this population [44]. While the most significant associations were with OT and IT [44], the correlation between IT and sunless tanning has not yet been made for adolescents and young adults. This behavior is consistent with combined IT and sunless tanning among adults [27, 45]. Some have hypothesized that engaging in both IT and ST is a consequence of commercial availability of both types of tanning in one, convenient location [35]. Given the use of these products among our surveyed population and their combination with UV tanning behaviors, more research is needed on the safety of sunless tanning product use regarding frequency and potential harmful effects associated with combining this behavior with other types of tanning.

The present study has many strengths, especially its large sample of over 2500 respondents, making it the largest college tanning study to date. Additionally, it examines a geographical population that has not been well-represented in the literature, as the majority of college tanning research has focused on Northeastern and Midwestern populations. It also identifies and quantities the use of multiple intentional tanning behaviors among this populations, while most work focuses on a single tanning behavior.

### Limitations

Most participants were residents of Alabama or the South (79.6%), therefore this sample is not necessarily representative of all college students throughout the United States. However, as noted above, this provides insight into a traditionally understudied and at-risk population. Due to gaps in the current literature, we are unable to determine the generalizability of these findings to the greater Gulf Coast region. While the survey response rate was approximately 25%, this rate is comparable to rates of other electronic surveys in similar populations [33]. As with all surveys, there is the risk of self-report, recall, and other bias.

## Conclusions

In this large survey of undergraduates from a Southern, coastal university we found significant engagement in tanning behaviors. More than half of the surveyed students engaged in OT and/or IT with a majority of tanners being white females. Further, there is an emerging population of ST, and again these tanners are predominantly white females. Of particular note is the high association between different tanning methods that indicates this population engages in multiple tanning behaviors. Combination tanning behaviors are not well understood and have not been well described in the literature, but this study suggests that these behaviors occur frequently. Given the literature associations between UV exposures and the risks associated with age of initiation and sex, our data suggest that white female tanners in the Southern U.S. may be at particular risk of developing skin cancer in the future and are prime candidates for interventions.

Future work in this area should focus on creating more standardized methodology and questions such as specific definitions of OT, IT, ST, and frequency to facilitate generalization and comparability of findings. New studies should also focus on the understudied trend of combination tanning (engaging in two or more types of tanning) demonstrated here with respect to predictors, long-term outcomes, and interventions targeting this type of behavior.

## Abbreviations

AOR: Adjusted odds ratio
AYA: Adolescents and young adults
CI: Confidence interval
IT: Indoor tanning
OR: Odds ratio
OT: Outdoor tanning
ST: Spray tanning
US: United States
UV: Ultraviolet
